# Influence of Precursor Nature on the Properties of Hydroxyapatite–Zirconia Nanocomposites

**DOI:** 10.3390/ma19030467

**Published:** 2026-01-24

**Authors:** Andreia Cucuruz, Cristina-Daniela Ghitulică, Daniela Romonti, Georgeta Voicu

**Affiliations:** 1 Department of Biomaterials and Medical Devices, Faculty of Medical Engineering, National University of Science and Technology Politehnica Bucharest, 1-7 Gh. Polizu Street, 011061 Bucharest, Romania; 2 Department of Science and Engineering of Oxide Materials and Nanomaterials, Faculty of Chemical Engineering and Biotechnologies, National University of Science and Technology Politehnica Bucharest, 1-7 Gh. Polizu Street, 011061 Bucharest, Romania; 3 Department of Applied Sciences, Faculty of Science, South East Technological University, R93 V960 Carlow, Ireland

**Keywords:** hydroxyapatite, zirconia, nanocomposites

## Abstract

This study explores the influence of precursor nature on the structural and mechanical characteristics of hydroxyapatite–yttria partially stabilized zirconia (HAp–YSZ) nanocomposites designed for biomedical applications. Precursor powders for obtaining these ceramic composites were synthesized via wet coprecipitation, using different calcium phosphate precursors: dibasic and monobasic ammonium phosphates for hydroxyapatite, and zirconyl chloride with yttrium acetate for YSZ. The dried precipitated powders were thermally treated at 600 °C and 800 °C and characterized by X-ray diffraction (XRD), thermal analysis (DTA–TG), transmission electron microscopy (TEM), and BET surface area measurements. The nanocomposites containing 70–90 wt.% HAp and 10–30 wt.% YSZ were sintered between 1000 °C and 1400 °C. Microstructural and physical properties were evaluated using scanning electron microscopy (SEM), open porosity, and compressive strength testing. Results revealed that precursor type and calcination temperature strongly affected crystallinity, particle size, and phase composition, influencing both porosity and mechanical strength of the final materials. An optimal sintering temperature of approximately 1200 °C was identified, balancing densification and phase stability. The findings demonstrate that controlling precursor chemistry and heat treatment enables fine-tuning of nanocomposite structure and performance, supporting their potential as bioactive, mechanically enhanced ceramics for orthopedic implant applications.

## 1. Introduction

Recent research on biocompatible ceramic nanocomposites, together with the integration of nanotechnology into biomedical research, has attracted considerable interest in the fields of tissue engineering and the development of materials with enhanced performance. The physical and chemical properties of ceramic nanocomposites enable the design of structures with advanced biomechanical properties while simultaneously achieving superior biological characteristics. Materials engineered at the nanoscale exhibit distinctive features, such as dimensional compatibility with biomolecules, high specific surface area, tunable physicochemical properties, and favorable biological responses, which have received significant attention in recent years [1,2].

A reference material for implant engineering applications is hydroxyapatite (HAp), owing to its excellent compatibility with hard tissues, as it represents their main mineral component [3,4]. Depending on the synthesis technique employed, hydroxyapatite may exhibit increased surface roughness, which contributes to enhanced osteoconductivity and facilitates tissue integration [5,6,7]. However, HAp is characterized by poor mechanical properties, including low strength and fracture toughness, which limit its use in load-bearing applications.

To improve these properties, recent studies have demonstrated that the incorporation of ceramic additives, such as alumina (Al_2_O_3_), zirconia (ZrO_2_), or titanium dioxide (TiO_2_), represents an effective strategy. Among these, zirconium oxide is a well-known material characterized by excellent biocompatibility and good stability in the physiological environment. Moreover, ZrO_2_ exhibits superior mechanical properties, including high wear resistance, toughness, and hardness [8,9,10,11,12,13]. Nevertheless, its bioinert nature constitutes the main limitation for bone substitution applications [14,15,16].

In this context, combining hydroxyapatite with zirconia particles leads to the development of ceramic nanocomposites with improved biomechanical properties, suitable for applications requiring enhanced mechanical performance [14,16,17].

The aim of this study was to synthesize and characterize ceramic nanocomposite materials based on hydroxyapatite and yttria-stabilized zirconia, in order to evaluate the influence of precursor nature, composition, and thermal treatments on the final structural and mechanical properties. The synthesis of the component powders was carried out via a wet chemical route using the coprecipitation method, employing different precursors for the phosphate phase: dibasic ammonium phosphate and monobasic ammonium phosphate. It was carried out to investigate the effect of phosphate species (HPO_4_^2−^ vs. H_2_PO_4_^−^) on precipitation kinetics and HAp microstructure (crystallinity/size), parameters known to be controlled by pH and precursor chemistry in wet synthesis; also these phosphates do not induce the substitution of foreign species in the HAp crystalline network, as is the case, for example, at use the alkaline or alkaline-earth phosphates [18,19,20]. The resulting nanocomposites contained variable proportions of HAp (70 and 90 wt.%) and ZrO_2_ (30 and 10 wt.%) and were sintered at temperatures of 1000 °C and 1400 °C to correlate processing parameters with microstructure, porosity, and mechanical behavior of the final materials.

## 2. Materials and Methods

### 2.1. Materials

**Figure 1:**
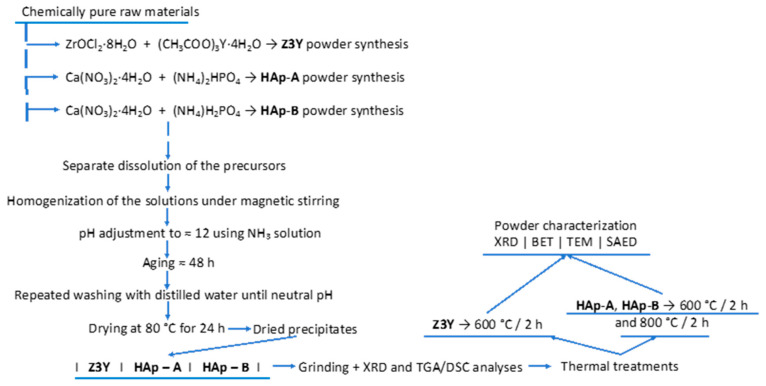
Schematic representation of the synthesis process of the component powders required for the fabrication of nanocomposites.

Within the scope of the study, the synthesis of the powder-form components required for the fabrication of nanocomposite ceramic materials was carried out using the coprecipitation method, according to the scheme presented in Figure 1, employing reagents of analytical grade purity.

ZrO_2_ powders doped with 3 mol% Y_2_O_3_ (Z3Y) were obtained by coprecipitation, using zirconyl chloride (ZrOCl_2_·8H_2_O, ≥97.0%, Sigma-Aldrich, Taufkirchen, Germany) and yttrium acetate ((CH_3_COO)_3_Y·4H_2_O, 99.99%, Sigma-Aldrich, Germany).

Hydroxyapatite powders were synthesized starting from calcium nitrate tetrahydrate (Ca(NO_3_)_2_·4H_2_O, ≥99.0%, Sigma-Aldrich, Germany) and dibasic ammonium phosphate ((NH_4_)_2_HPO_4_, ACS reagent, ≥98%, Sigma-Aldrich, Germany) for the HAp-A variant, and monobasic ammonium phosphate ((NH_4_)H_2_PO_4_, ACS reagent, ≥98%, Sigma-Aldrich, Germany) for the HAp-B variant.

### 2.2. Powder Synthesis

After the separate dissolution of the precursors, the solutions were homogenized under continuous stirring, followed by adjustment of the pH of the final solution to approximately 12, in order to ensure a basic environment favorable to the coprecipitation process, by the addition of an ammoniacal solution.

The resulting precipitates were aged at room temperature, in air, for 2 days to allow precipitate maturation and completion of the chemical reactions, thereby ensuring adequate chemical homogeneity.

The obtained precipitates were filtered and washed with distilled water to remove residual ammonia until a nearly neutral pH was reached. Subsequently, the precipitates were dried at 80 °C for 24 h. After drying, the powders were ground in an agate mortar and analyzed by X-ray diffraction (XRD) and simultaneous thermal analysis. The powders were then calcined, namely the Z3Y powders at 600 °C for 2 h, and the HAp-A and HAp-B powders at 600 °C and 800 °C for 2 h. After the thermal treatments, the powders were characterized by various techniques (Figure 1), including X-ray diffraction (XRD), Brunauer–Emmett–Teller specific surface area analysis (BET), transmission electron microscopy (TEM), and selected area electron diffraction (SAED).

### 2.3. Fabrication of Nanocomposite Ceramic Materials

**Figure 2:**
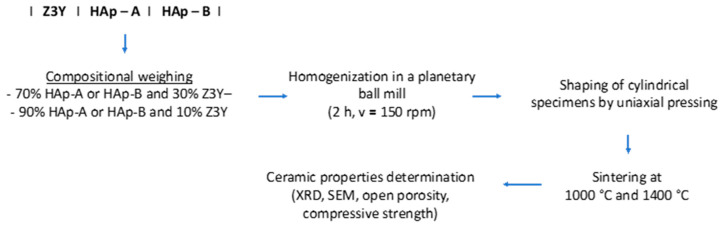
Processing steps for obtaining nanocomposite ceramic materials.

Nanocomposite ceramic materials were fabricated according to the scheme presented in Figure 2, with compositions containing different proportions of hydroxyapatite (HAp-A and HAp-B), namely 70 and 90 wt.%, while the remaining 30 and 10 wt.% consisted of zirconia powders (Z3Y), respectively.

The powders were homogenized by planetary ball milling for 2 h at a rotational speed of 150 rpm (ball-to-powder ratio of 10:1 (*w*/*w*) using corundum balls and jar), then shaped by uniaxial pressing (ϕxh = 10.13 × 10, pressure = 2 t, corresponding to an applied pressure of aprox. 250 MPa), followed by thermal sintering treatments carried out at temperatures of 1000 °C and 1400 °C with a holding time of 2 h, to obtain dense nanocomposite ceramic materials.

The precursor powders were characterized using various experimental techniques. Thus, compositional characterization was performed by X-ray diffraction (XRD) and simultaneous thermal analysis (DTA–TG), while the morphostructural and dispersive characterization of the ceramic powders was carried out using transmission electron microscopy (TEM) and Brunauer–Emmett–Teller (BET) analysis. In addition to porosity, the BET analyses were also used to estimate the average particle size according to the following Equation (1) [17]:

(1) $$D=\;\frac{6}{\left(S\times \;d\right)}$$

where D is the average particle size (nm), *S* is the specific surface area (m^2^/g), and *d* is the theoretical density of the material (g/cm^3^).

The nanocomposites were compositionally characterized by X-ray diffraction (XRD) and microstructurally characterized by scanning electron microscopy (SEM). The characteristic ceramic properties (e.g., open porosity) were determined using the Archimedes method (Arthur), while the compressive mechanical strength was evaluated using a universal mechanical testing machine.

### 2.4. Characterization Techniques

XRD analysis was performed using a Shimadzu XRD 6000 diffractometer (Shimadzu, Kyoto, Japan), equipped with Ni-filtered CuKα radiation (λ = 1.5406 Å).

DTA–TG analysis was carried out in an air atmosphere using a Shimadzu DTG-TA-50H instrument (Shimadzu, Kyoto, Japan), with a heating rate of 10 °C/min, up to a maximum temperature of 1000 °C.

The morphology of the samples was analyzed by scanning electron microscopy (SEM) using a HITACHI S2600N scanning electron microscope, with a resolution of 4 nm (Hitachi High-Tech Corporation, Tokyo, Japan); the nanocomposite ceramic materials were coated with a thin gold layer prior to analysis.

In order to estimate both the average particle size and the crystallinity degree, TEM-SAED/HRTEM investigations were performed using a Tecnai TM G2 F30 S-TWIN high-resolution transmission electron microscope (HR-TEM) (Thermo Fisher Scientific, Waltham, MA, USA) equipped with STEM–HAADF detector, EDX, and EELS.

The specific surface area and pore size were determined by Brunauer–Emmett–Teller (BET) analysis using a Micromeritics Gemini V2, model 2380 analyzer (Micromeritics Instruments Corporation, Norcross, GA, USA).

The characteristic ceramic properties, namely density and open porosity, were determined using the Archimedes method (Arthur) [21], while the compressive mechanical strength was evaluated using a universal mechanical testing machine (Walter Bai AG Testing Machine LFM 50 Kn, Walter + Bai AG, Löhningen, Switzerland).

## 3. Results and Discussion

### 3.1. Characterization of Precursor Powders Used for the Fabrication of Nanocomposite Ceramic Materials

The composition and degree of crystallinity of the dried precipitates were evaluated by X-ray diffraction (XRD) using a Shimadzu XRD 6000 diffractometer (Shimadzu, Kyoto, Japan), equipped with Ni-filtered CuKα radiation (λ = 1.5406 Å).

**Figure 3:**
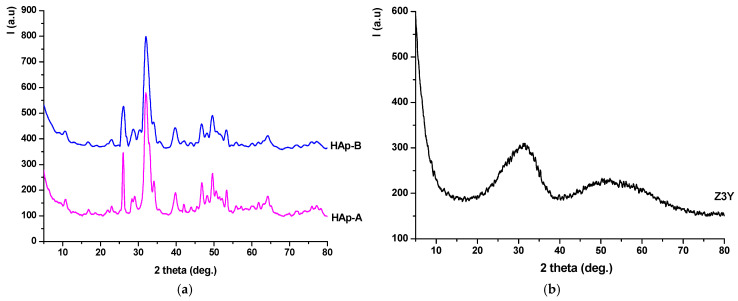
X-ray diffraction patterns of the precipitates dried at 80 °C: (**a**) HAp-A; HAp-B; (**b**) Z3Y.

**Figure 4:**
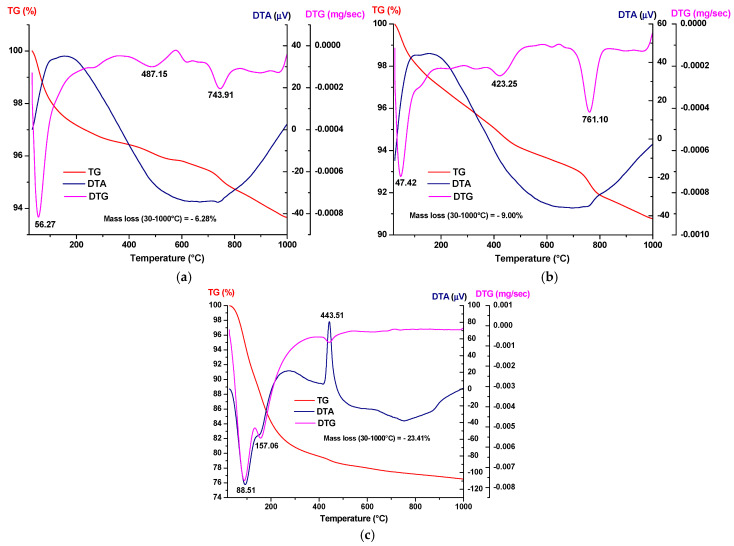
Simultaneous thermal analysis of the precipitates dried at 80 °C: (**a**) HAp-A; (**b**) HAp-B; and (**c**) Z3Y.

The dried precursor powders were ground and analyzed by X-ray diffraction (Figure 3) and simultaneous thermal analysis (Figure 4).

From Figure 3a, it can be observed that the dried HAp-A and HAp-B precipitates contain hydroxyapatite as the crystalline mineralogical phase, in accordance with JCPDS 084-1998, as evidenced by the characteristic diffraction peaks. It should also be noted that, in the case of the HAp-B sample, the intensity ratio of the diffraction peaks remains approximately the same as for HAp-A; however, the peaks are broader and less intense, suggesting a smaller crystallite size. The dried Z3Y precipitate (Figure 3b) exhibits a pronounced amorphous character, with the diffractogram showing two distinct halos in the 20–40° and 45–60° 2θ ranges.

From the simultaneous thermal analysis presented in Figure 4, the following can be observed:

- For all three dried precipitates, weight losses are recorded in the temperature range of 30–1000 °C, amounting to 6.28% for the HAp-A precipitates and 9.01% for the HAp-B precipitates, while the Z3Y precipitates exhibit a weight loss of 23.41%, accompanied by various thermal effects visible on the DTA curves;
- For the phosphate precipitates, the endothermic effect observed at approximately 50 °C indicates water evaporation processes; at around 423–487 °C, the decomposition of Ca(OH)_2_ occurs, while at 743–761 °C, the decomposition of CaCO_3_ formed due to accidental carbonation by atmospheric CO_2_ takes place;
- The Z3Y sample exhibits two endothermic effects at 88 °C and 157 °C and one exothermic effect at 443 °C on the DTA curve, all accompanied by mass loss; the two endothermic effects are attributed to the loss of physically adsorbed water, while the exothermic effect accompanied by mass loss is attributed to the combustion of residual organic components.

Based on these analyses, in order to eliminate gas-generating processes and obtain the final powders, the dried phosphate precipitates were thermally treated at 600 °C for 2 h and 800 °C for 2 h, while the zirconia-based precipitates were treated at 600 °C for 2 h.

From the standpoint of morpho-textural and dispersive characteristics, the dried precipitates and thermally treated powders were analyzed by X-ray diffraction (XRD), Brunauer–Emmett–Teller (BET) analysis, and transmission electron microscopy (TEM). In addition, selected area electron diffraction (SAED), performed during TEM analysis, was used to assess the degree of crystallinity and phase composition.

**Figure 5:**
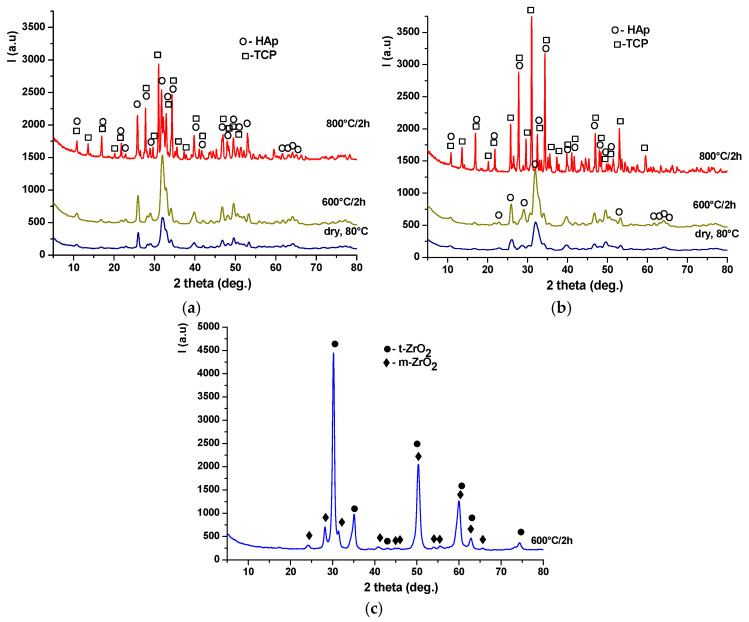
X-ray diffraction patterns of the precipitates dried at 80 °C and thermally treated at 600 °C for 2 h and 800 °C for 2 h: (**a**) HAp-A; (**b**) HAp-B; and (**c**) Z3Y.

The X-ray diffraction patterns of the dried phosphate precipitates and the thermally treated powders (Figure 5a,b) show that up to 600 °C, the powders contain hydroxyapatite (HAp) as the main crystalline phase, in accordance with JCPDS 084-1998, while after treatment at 800 °C for 2 h, the materials consist of a mixture of hydroxyapatite and tricalcium phosphate (β-TCP), in agreement with JCPDS 009-0169.

The degree of crystallinity of TCP is higher in the HAp-B powder composition than in HAp-A, most likely due to the promotion of the HAp-to-TCP transformation, considering that in this case, the HAp phase up to 600 °C exhibits lower crystallinity and smaller crystallite size (Figure 5b). For the zirconia-based Z3Y precipitate (Figure 5c), it can be observed that thermal treatment at 600 °C for 2 h leads to the formation of a crystalline mixture consisting of tetragonal zirconia (t-ZrO_2_), according to JCPDS 079-1769, and monoclinic zirconia (m-ZrO_2_), according to JCPDS 083-0944.

The BET specific surface area data and particle size (D) values are summarized in Table 1.

It can be observed that the dried precipitates and the powders thermally treated up to 600 °C exhibit particle sizes within the nanomaterial range; however, upon thermal treatment of the phosphate precipitates at 800 °C, the average particle size increases significantly. Additionally, the specific surface area of the powders is found to vary inversely with particle size, decreasing as the thermal treatment temperature increases. In the case of the zirconia-based powders, thermal treatment leads to an increase in particle size, which nevertheless remains within the nanometric range.

**Figure 6:**
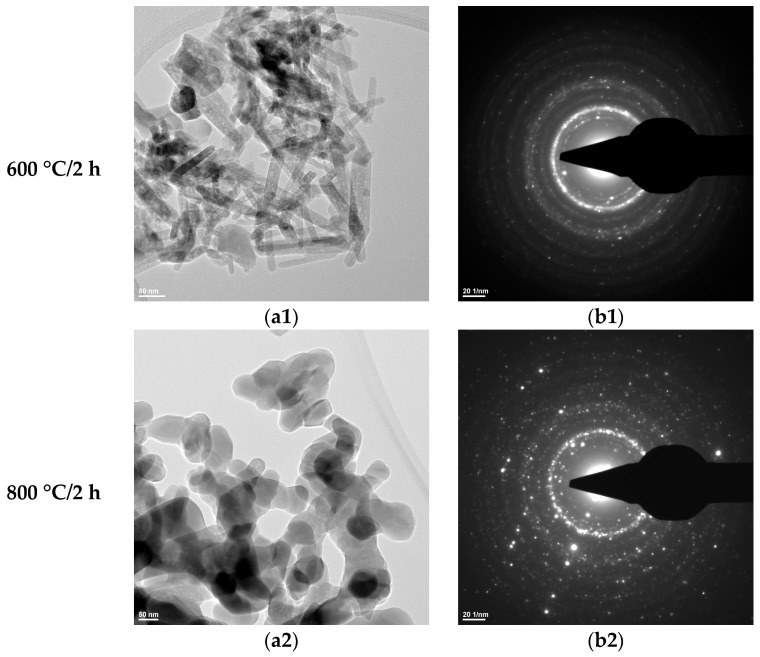
Transmission electron microscopy analysis (TEM: (**a1**,**a2**); SAED: (**b1**,**b2**)) of the HAp-A powder thermally treated at 600 °C for 2 h and 800 °C for 2 h.

**Figure 7:**
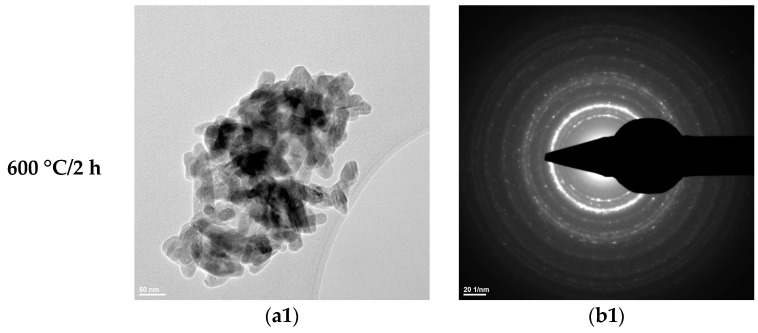

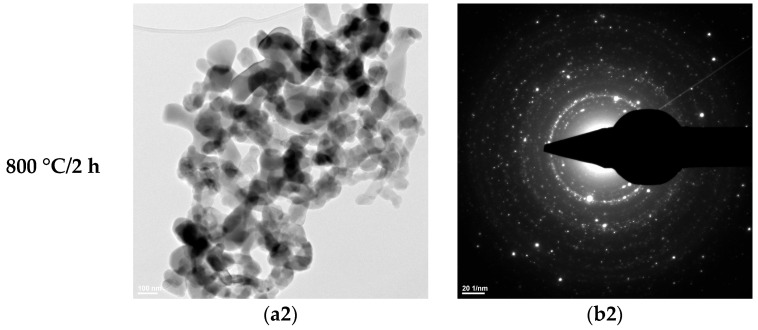
Transmission electron microscopy analysis (TEM: (**a1**,**a2**); SAED: (**b1**,**b2**)) of the HAp-B powder thermally treated at 600 °C for 2 h and 800 °C for 2 h.

**Figure 8:**
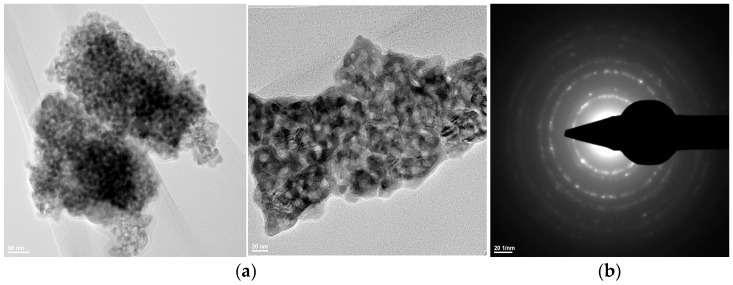
Transmission electron microscopy analysis (TEM: (**a**); SAED: (**b**)) of the Z3Y powder thermally treated at 600 °C for 2 h.

From the transmission electron microscopy (TEM) analyses (Figure 6, Figure 7 and Figure 8), the following can be observed:

– Hydroxyapatite exhibits a rod-like morphology with dimensions of 100–200 nm, as well as a quasi-spherical morphology with sizes in the range of 5–10 nm (Figure 6(a1) and Figure 7(a1));
– Tricalcium phosphate displays an elongated, ellipsoidal, slightly deformed particle morphology, with dimensions of 100–200 nm (Figure 6(a2) and Figure 7(a2));
– Zirconium oxide stabilized with 3 mol% yttrium oxide and thermally treated at 600 °C for 2 h exhibits particle sizes below 10 nm and a predominantly spherical morphology (Figure 8).

The SAED data (Figure 6(b1,b2), Figure 7(b1,b2), and Figure 8b) indicate the polycrystalline nature of both the dried precipitates and the thermally treated powders. In the case of the phosphate-based precipitates, an increase in the thermal treatment temperature leads to an enhancement of the degree of crystallinity.

Therefore, these transmission electron microscopy data are consistent with the recorded X-ray diffraction patterns and with the average particle size values calculated based on the BET analyses.

### 3.2. Synthesis and Characterization of HAp–ZrO_2_ Nanocomposite Ceramic Materials

Table 2 presents the compositions of the nanocomposite ceramic materials obtained from HAp-A/HAp-B powders thermally treated at 600 °C for 2 h and 800 °C for 2 h, respectively, and Z3Y powder thermally treated at 600 °C for 2 h. The Z3Y mass fraction was 10% and 30%, while the thermal treatment of the nanocomposite ceramic materials, according to the scheme shown in Figure 2, was carried out at 1000 °C for 2 h and 1400 °C for 2 h.

**Figure 9:**
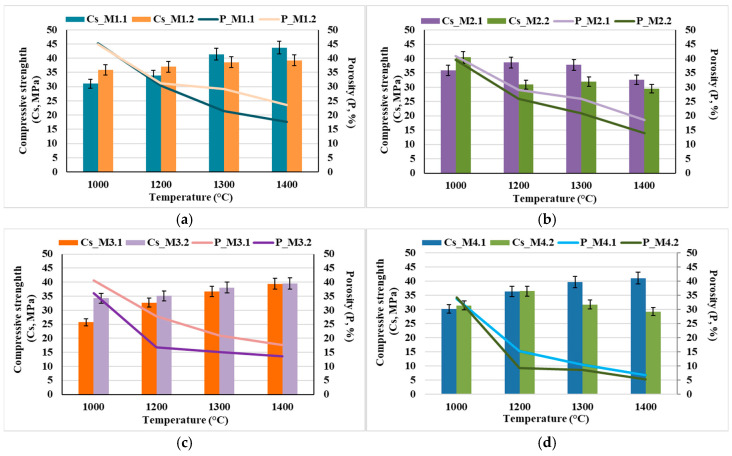
Characteristic physical properties of the nanocomposite ceramic materials: open porosity and compressive strength as a function of the sintering temperature: (**a**) M1.1 and M1.2; (**b**) M2.1 and M2.2; (**c**) M3.1 and M3.2; (**d**) M4.1 and M4.2.

The results obtained for the characteristic physical properties of the nanocomposite ceramic materials (open porosity and compressive strength) as a function of the sintering temperature are presented in Figure 9.

It can be observed that the mechanical strength increases concomitantly with the decrease in open porosity; however, it is also influenced by particle size—implicitly by the calcination temperature—as well as by the HAp content of the composite and the sintering temperature. Thus, the specimens obtained from nanopowders (resulting from the calcination of dried precipitates at 600 °C for 2 h) exhibit higher compressive strength values (Figure 9a,c) compared to those prepared from powders with particle sizes in the micrometric range (obtained by calcination of the dried precipitates at 800 °C for 2 h), even under conditions of higher open porosity (Figure 9a vs. Figure 9b and Figure 9c vs. Figure 9d, respectively). This behavior can most likely be attributed to the significant influence of pore size on the mechanical response, in the sense that smaller pores, even when present in a higher proportion, have a less detrimental effect on mechanical behavior than larger pores, even if the latter are present in lower amounts [22,23,24,25].

**Figure 10:**
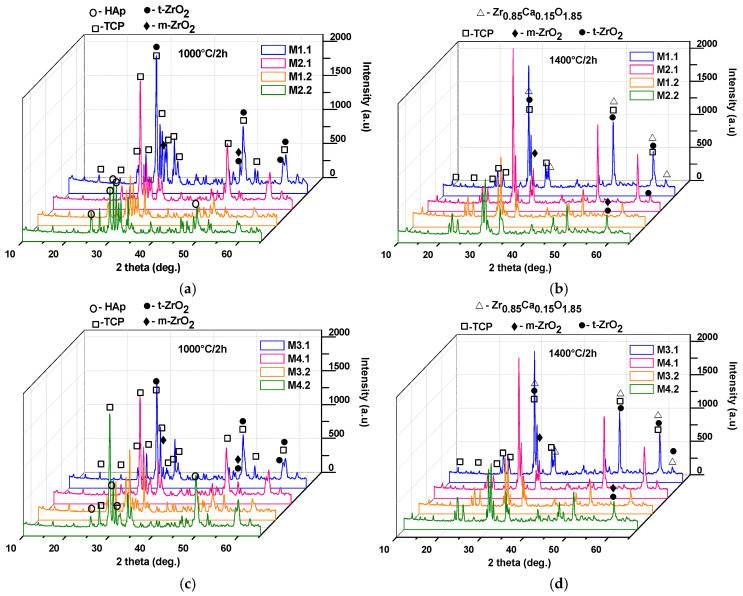
X-ray diffraction patterns of the nanocomposite ceramic materials as a function of the sintering temperature (1000 °C/2 h: (**a**,**c**); 1400 °C/2 h: (**b**,**d**)).

**Figure 11:**
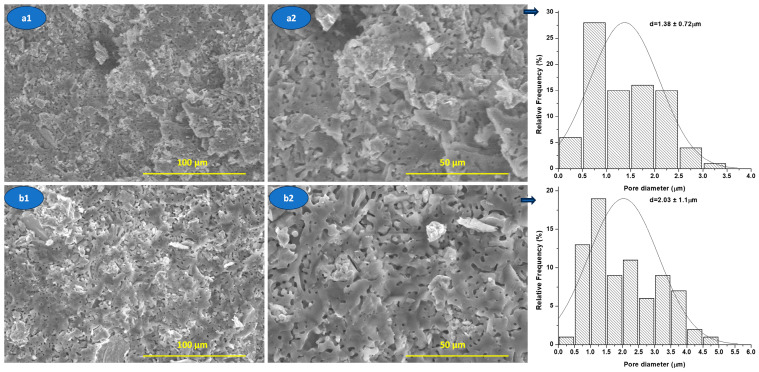
SEM images of the nanocomposite ceramic materials M1.1 (**a1**,**a2**) and M3.1 (**b1**,**b2**) sinterised at 1400 °C/2 h.

Additionally, it can be observed that, in general, the mechanical properties improve with increasing thermal treatment temperature; however, there exists an optimal temperature up to which these properties increase, beyond which they may either decrease (e.g., for samples M2.2 and M4.2) or remain nearly constant (e.g., for samples M1.2 and M2.2). This behavior can be correlated with the phase composition and microstructural characteristics of the nanocomposite ceramic materials, as evidenced in Figure 10 and Figure 11.

Thus, as shown in Figure 10, the use of powders calcined at 600 °C and 800 °C (see Figure 5a,b), followed by an increase in the sintering temperature from 1000 °C to 1400 °C, leads to the transformation of the HAp phase (JCPDS 084-1998) into tricalcium phosphate (at 1000 °C: β-TCP, JCPDS 009-0169; at 1400 °C: α-TCP, JCPDS 029-0359), along with an increase in the degree of crystallinity. Additionally, the diffractograms reveal characteristic peaks of tetragonal zirconia (t-ZrO_2_), according to JCPDS 079-1769, and monoclinic zirconia (m-ZrO_2_), according to JCPDS 083-0944. In addition, at 1400 °C, interferences specific to a calcium zirconate can be observed (Zr_0.85_Ca_0.15_O_1.85_; JCPDS 084-1827), which, according to the literature, does not induce negative effects from the point of view of biological behavior [26,27].

From Figure 11, it can be seen that nanocomposite ceramic materials are characterized by porosity, which is generated, most probably, by decomposition of HAp during sintering treatment, according to mechanical behavior (see Figure 9).

The SEM images (Figure 11) highlight a porous microstructure with predominantly open pores, relatively evenly distributed in the ceramic matrix. The pores mainly exhibit an irregular, interconnected morphology, suggesting that they result from processes associated with sintering, such as the release of gases during HAp dehydroxylation.

The analysis of the pore size distribution indicates an average pore size of approximately 1.38 ± 0.72 μm for the M1.1 sample and 2.03 ± 1.11 μm for the M3.1 sample, confirming the existence of clear differences in the porous architecture between the two systems. Samples with larger pores and a wider distribution (M3.1 sample) exhibit increased pore connectivity, which favors open porosity, but at the same time leads to the concentration of local stresses, negatively affecting mechanical strength.

This observation is consistent with the mechanical results reported in the manuscript (see Figure 9), where a decrease in compressive strength correlates with an increase in pore size and connectivity, not just the total porosity fraction.

## 4. Conclusions

In this study, hydroxyapatite–yttria-stabilized zirconia (HAp–YSZ) ceramic nanocomposites were successfully synthesized via a wet chemical coprecipitation route, using different phosphate precursors, followed by controlled thermal treatments. The influence of precursor nature, calcination temperature, composition, and sintering conditions on the structural, microstructural, and mechanical properties of the resulting materials was systematically investigated.

X-ray diffraction, thermal analysis, BET measurements, and electron microscopy revealed that both the precursor type and the calcination temperature strongly affect phase composition, crystallinity, particle size, and specific surface area of the powders. Calcination at 600 °C resulted in nanometric powders with high specific surface area, while higher calcination temperatures (800 °C) promoted grain growth and partial transformation of hydroxyapatite into tricalcium phosphate (β-TCP).

The mechanical behavior of the nanocomposite ceramics was found to be closely correlated with microstructural features such as particle size, pore size, phase composition, and degree of densification. Specimens fabricated from nanopowders exhibited superior compressive strength compared to those obtained from micrometric powders, even at higher levels of open porosity, highlighting the dominant role of pore size over total porosity in determining mechanical performance.

An increase in sintering temperature generally led to improved mechanical properties; however, an optimal sintering temperature was identified, approximately 1200 °C, beyond which further temperature increase resulted in phase transformations (e.g., β-TCP → α-TCP) and microstructural changes that could limit or even reduce mechanical performance.

Overall, the results demonstrate that careful control of precursor chemistry and thermal processing enables effective tailoring of the structure and properties of HAp–YSZ nanocomposites. These materials combine bioactivity with enhanced mechanical performance, making them promising candidates for biomedical applications, particularly in load-bearing orthopedic and dental implant systems.

## Figures and Tables

**Table 1:** BET specific surface area and particle size (D) of the synthesized powders.

Sample	HAp-A 80 °C	HAp-A 600 °C	HAp-A 800 °C	HAp-B 80 °C	HAp-B 600 °C	HAp-B 800 °C	Z3Y 80 °C	Z3Y 600 °C
S_BET_ (m^2^/g)	84.70	28.04	10.61	64.52	35.93	6.35	174.64	42.26
D (nm)	22.4	67.7	182.5	29.5	52.8	307.7	5.6	23.3

**Table 2:** Compositions of HAp-A/HAp-B powders, thermally treated at 600 °C for 2 h and 800 °C for 2 h, respectively, and Z3Y powder, thermally treated at 600 °C for 2 h, used for the fabrication of nanocomposite ceramic materials.

Composite Code	M1.1 (A600)	M1.2 (A600)	M2.1 (A800)	M2.2 (A800)	M3.1 (B600)	M3.2 (B600)	M4.1 (B800)	M4.2 (B800)
HAp (%)	70	90	70	90	70	90	70	90
Z3Y (%)	30	10	30	10	30	10	30	10

## Data Availability

The original contributions presented in this study are included in the article. Further inquiries can be directed to the corresponding author.
